# Predicting differential effects of sex on therapeutic benefit and limitations in Alzheimer’s disease drug development using animal models

**DOI:** 10.1002/alz70859_098665

**Published:** 2025-12-25

**Authors:** Stacey J Sukoff Rizzo

**Affiliations:** ^1^ University of Pittsburgh School of Medicine, Pittsburgh, PA USA

## Abstract

**Background:**

There has been an overwhelming need to increase the predictive validity of preclinical studies for safety and efficacy in Alzheimer’s disease (AD) using animal models; and importantly to inform differential therapeutic benefits and limitations across sexes. The Preclinical Testing Core (PTC) of the MODEL‐AD consortium has established resources for the research community for unbiased screening of potential therapeutics with prioritization of translational outcome measures, and powered to detect effects of sex in mouse models.

**Methods:**

For each potential drug being evaluated, the PTC selects a mouse model specific to the compound’s mechanism of action being investigated that best recapitulates the human disease signature and biology, and that can enable and inform translational in vivo target engagement and a pharmacokinetic (PK) and pharmacodynamic (PD) relationship. All studies not only include both males and females, but are powered to detect effects of sex. Initial PK studies capture a broad dose range which are used to inform differential PK/PD across sex, such that the experimental design for long‐term chronic studies captures sex‐specific target engagement and exposure levels. Compounds advancing through the pipeline that have been de‐risked and demonstrate pharmacodynamic effects that may predict efficacy can be advanced to more sophisticated cognitive studies including translational touchscreen platforms that can be also employed similarly in non‐human primates. This screening strategy has been used to characterize a variety of mechanisms of action in 5XFAD mice including the small molecules levetiracetam and verubecestat, and the murinized/chimeric monoclonal antibody aducanumab.

**Results:**

Using the unbiased and rigorous screening approaches of the PTC, we have identified divergent effects of sex on PK measures (including plasma and brain exposures), efficacy (e.g., amyloid lowering) and behavioral effects (including side‐effects) in all compounds tested. These data in the mice were consistent with clinical data demonstrating differential effects of sex on therapeutic benefits and limitations reported for each of these compounds

**Conclusions:**

Using rigorous and unbiased screening approaches that are powered to detect effects of sex in animal models can be used to predict differential effects of sex on therapeutic benefit and limitations of novel compounds being investigated for the treatment of AD.

